# Association of laboratory parameters with viral factors in patients with hepatitis C

**DOI:** 10.1186/1743-422X-8-361

**Published:** 2011-07-21

**Authors:** Bushra Ijaz, Waqar Ahmad, Fouzia T Javed, Sana Gull, Muhammad T Sarwar, Humera Kausar, Sultan Asad, Shah Jahan, Saba Khaliq, Imran Shahid, Aleena Sumrin, Sajida Hassan

**Affiliations:** 1 Applied and Functional Genomics lab, Centre of Excellence in Molecular Biology, University of the Punjab, Lahore, Pakistan; 2 Department of Pathology, Jinnah Hospital, Lahore, Pakistan

## Abstract

**Background and Aims:**

HCV infection may lead to hepatic fibrosis. In this study, we tried to determine whether there is any correlation of HCV genotypes and viral load to the clinical parameters such as ALT, AST, ALP, bilirubin, Hb level, patient's age and gender; and then correlated this association with disease progression in liver biopsy samples.

**Methods:**

In cross-sectional and observational study, 6048 serum HCV RNA positive patients were chosen. The study consists of 53 months from March 2006 to September 2010. Patients were divided into three cohorts to validate our data. Statistical analysis and correlation of lab parameters with viral factors was determined by using SPSS version 16.

**Results:**

The most prevalent genotype was 3 (70.9%) followed by 1 (13.3%) and 4 (7.4%), collectively. During Univariate analysis, in all cohorts; serum bilirubin, ALP, ALT and AAR showed significant correlation with genotypes, however multivariate analysis showed that all genotypes except 4a have no association with host biochemical markers. Disease progression was also independent of all genotypes. Serum ALP, ALT, bilirubin and viremea levels were significantly elevated in patients with genotype 4a. Viral load showed negative association with serum bilirubin (*r *= -0.112, *P *= 0.000) and ALP levels (*r *= -0.098, *P *= 0.000). We observed positive correlation of ALP and bilirubin levels, while negative associations of viral load with HCV liver disease progression.

**Conclusion:**

Disease progression seems independent of the genotypes. Relationship between ALP and bilirubin with viral load may be an attractive marker to guess disease progression in patients with hepatitis C.

## Introduction

Approximately 3% of the world's populations, (more than 350 million people) and about, 10 million people in Pakistan [1] are chronically infected with hepatitis C virus (HCV). HCV is the main cause of liver fibrosis, cirrhosis and hepatocellular carcinoma (HCC) in a substantial number of patients [2]. Due to considerable sequence diversity HCV is classified into a series of genotypes showed distinct geographical and frequency distribution across the whole world [3-5].

In patients infected with HCV, clinical findings, genotypes and viral load are strong predictors for the outcome of antiviral therapy [6,7]. The most prevalent genotype in Pakistan is 3a followed by 3b and 1a [8]. Due to high prevalence of genotype 3a in Pakistan; HCV genotyping is not recommended in HCV infected patients routinely by Pakistan's society of Gastroenterology [9]. Secondly, due to poverty and cost of genotyping test, many patients do not agree for this test. Nevertheless, genotyping is important because it not only provides information as to strain variation and potential association with disease severity but is also related to the possibility of treatment response [10]. It is reported that treatment with interferon is more effective in patients with genotypes 2 and 3 than in patients infected with genotypes 1 and 4 [11]. An assessment of the disease development based on clinical findings is still critical for patients infected with HCV. Several authors tried to correlate viral and host biochemical factors like genotype, viral load, ALT, AST, bilirubin etc with each other as well as with liver damage, but no clear conclusions were formed [12-16].

In the present study, we investigated the correlation of several clinical findings like Hb, bilirubin, ALT, ALP and AST levels, and AAR with viral factors (viral load and genotypes) in patients infected with HCV; and their outcome with fibrosis stages. We collected the HCV positive samples and observed the best relation of serum markers and viral factors and assess this relation in liver histological grading for disease progression.

## Patients and methods

### Patients

Patients of this study were the people referred to Pathology department, Jinnah Hospital and Mayo Hospital Lahore; and Liver Centre Faisalabad Pakistan, for biochemical and serological tests. This analytical study was carried out from March 2006 to September 2010 in collaboration of National Centre of Excellence in Molecular Biology, University of the Punjab, Lahore, Pakistan. Patients were divided into three cohorts, (i) 2006-08, (ii) 2008-09 and (iii) 2009-10; first as initial cohort and later two as validation cohort to find appropriate relationship between viral and host serum markers. Blood samples (10 mL) were collected from each patient and tested for anti-HCV antibody by ELISA (Abbot Laboratories) at Jinnah Hospital Lahore. Patients with positive serology and/or positive test for HCV alone and no evidence of liver failure were included in this study. Patients who were not keen to give informed consent, not able to make follow-up visits and not willing to undergo genetic testing and not allow samples to be stored for future research were excluded from the study. Accordingly, thus, a total of 6048 HCV-RNA positive patients were identified. The routine liver function tests (LFTs), Hb level and direct bilirubin were estimated for each patient in the hospital laboratory by using commercially available Hitachi-7600 series analyzer. Questionnaire (including their personal, lab tests and demographical information) was prepared for patients who came for HCV initial screening and further genotyping and viral load quantification. Informed consents were (containing permission to do liver biopsy, procedure of liver biopsy and the possible risks associated with liver biopsy were mentioned) obtained from those patients who were willing to do so. Only patients aged 18 or above were considered for liver biopsy samples. Patients less than 18 years were only included in genotyping and biochemical data analysis etc. Regarding consent obtained from under 18 years children, parents signed and filled the questionnaire. The study was approved by the institutional ethical committee.

### HCV viral assays

HCV viral detection and genotyping was carried out at the Department of Pathology, Jinnah Hospital, Lahore, Mayo Hospital, Lahore and Liver Center, Faisalabad, Pakistan. QIAamp viral RNA extraction kit (Qiagen, USA) was used to extract HCV RNA from serum (150 μl) according to the manufacturer's protocol. The extracted RNA was reverse transcribed to cDNA using Moloney murine leukemia virus (MmLV) followed by PCR at 5`UTR non-coding region of HCV genome using primers described by Chan et al. 1992 [17]. After qualitative PCR analysis, Qiagen HCV RG RT-PCR assay was used for the quantification of viral RNA. Briefly 10ul of the extracted RNA was mixed with PCR mix and fluorescent probes and quantified on Rotor-gene Real-Time PCR machine (USA), amplification was detected after each replicating cycle as described by manufacturer protocol. The lower limit of detection for this assay is 1000 IU/ml. Invader HCV genotyping assay (Third wave technology, USA) was used for genotyping analysis. HCV RNA (100ng) was reverse transcribed to cDNA using 200U of MmLV (Invitrogen, USA). For genotyping assay, 2 μl of the cDNA was amplified and assay for 12 different HCV types was performed.

### Histological evaluation of biopsy samples

METAVIR scoring system was used for the histological evaluation of 157 paraffin-embedded liver specimens at Pathology Department Jinnah Hospital, Lahore [18]. Liver biopsies were evaluated by two independent pathologists without former information to patient's history. Liver histological staging was based on five degrees of fibrosis: as F0 (no fibrosis), F1 (mild fibrosis without septa) F2 (moderate fibrosis with few septa), F3 (severe fibrosis with numerous septa without cirrhosis) and F4 (cirrhosis). These stages were further grouped as F0-F1 (no/minimal fibrosis), F2-F3 (advanced fibrosis) and F4 (cirrhosis).

### Viral factors association with host biochemical factors

Viral factors like genotype and viral load were correlated with host biochemical conditions like ALT, AST, ALP, bilirubin and Hb level. We also checked this correlation in different fibrosis stages.

### Statistical analysis

Statistical analysis was performed using the statistical package for social studies (SPSS) version 16 for windows. Student t-test and Chi-square tests were applied to evaluate differences in proportions. *P *value <0.05 was considered significant. Univariate analysis includes the variables age, sex, Hb level, bilirubin, ALT, AST, ALP and viral load. Age, sex and genotypes were taken as independent categorical factors. Multiple regression analysis was used to evaluate independent associations between HCV genotypes and biochemical values. The relationship between serum markers and viral load was analyzed by Spearman's correlation for non-parametric data.

## Results

### Prevalence of HCV infection

Patients were divided in three cohorts. Overall, out of 6048 patients, 3066 (50.7%) were male while 2982 (49.3%) were female. The first cohort was observational cohort while, the successive years data was validation cohort. The mean age of patients was 37.40 ± 10.9 years (range 6-75), while 3815 (63.1%) patients were ≤ 40 year of age. Table 1 briefly outlined patients' data for each cohort.

**Table 1:** Demographic and biochemical characteristics of patients in initial and validation cohorts

Variables	Initial Cohort 2006-08 (n = 3160)>	Validation Cohort 1 2008-09 (n = 1364)	Validation Cohort 2 2009-10 (n = 1524)	Overall 2006-10 (n = 6048)
**Gender**				
Male	1515 (47.9%)	718 (52.6%)	833 (54.7%)	3066 (50.7%)
Female	1645 (52.1%)	646 (47.4%)	691 (45.3%)	2982 (49.3%)
**Age**				
Mean	37.08 ± 10.33	37.15 ± 11.65	38.29 ± 11.361	37.40 ± 10.91
Range	6-75	12-71	10-75	6-75
**Age Group**				
<40	2119 (67.1%)	832 (61.0%)	864 (56.7%)	3815 (63.1%)
>40	1041 (32.9%)	532 (39.0%)	660 (43.3%)	2233 (36.9%)
**Biochemical Markers (All values in median (Range)**				
Viral titer (IU/mL)	1.0 × 10^7 ^(1340-8.5 × 10^8^)	3.20 × 10^5 ^(10000-4.0 × 10^8^)	1.8 × 10^7 ^(10000-4.0 × 10^8^)	2.0 × 10^6 ^(1340-8.5 × 10^8^)
Hb level(mg/dl)	13.1 (9.8-16.7)	14.0 (13.1-16.9)	11.4 (8.8-15.6)	13.1 (8.8-16.9)
Bilirubin (mg/dl)	0.80 (0.1-1.3)	0.80 (0.3-1.2)	0.80 (0.1-1.9)	0.80 (0.1-1.9)
ALP (IU/mL)	185.0 (85-421)	165.0 (30-396)	165.5 (30-392)	176.0 (24-421)
ALT (IU/mL)	58.0 (14-238)	57.0 (13-311)	59.0 (11-308)	58.0 (11-311)
AST (IU/mL)	56.0 (13-230)	55.0 (16-218)	57.0 (19-236)	56 (13-236)
AAR	0.96 ± (0.155.75)	0.96 (0.07-7.50)	0.98 (0.06-9.09)	0.96 (0.06-9.09)
**Genotypes**				
1	365 (11.6%)	206 (15.1%)	231 (15.2%)	802 (13.3%)
2	31 (1.0%)	27 (2.0%)	55 (3.6%)	113 (1.9%)
3	2359 (74.7%)	934 (68.5%)	994 (65.2%)	4287 (70.9%)
4	224 (7.1%)	89 (6.5%)	133 (8.7%)	446 (7.4%)
5	28 (0.9%)	0	3 (0.2%)	31 (0.5%)
6	19 (0.6%)	4 (0.3%)	3 (0.2%)	26 (0.4%)
Mix	96 (3.0%)	46 (3.4%)	55 (3.6%)	197 (3.3%)
Undefined	38 (1.2%)	58 (4.3%)	50 (3.3%)	146 (2.4%)

### Genotype distribution among patients

**Figure 1:**
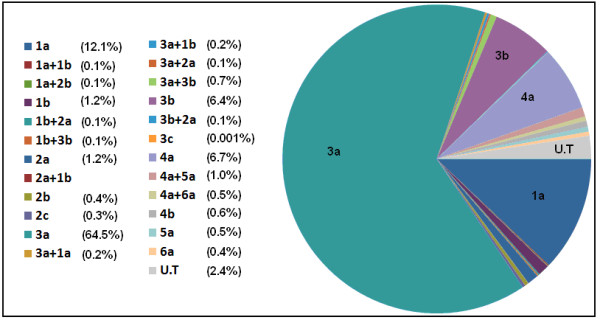
**Distribution of genotypes subtypes and mix genotypes in patients**. Genotype 3a was most frequent followed by 1a, 4a and 3b.

Based on weighted analysis of patients infected with HCV in all cohorts, the most frequently detected genotype was 3 (70.9%), with predominant subtype 3a (64.5%) and 3b (6.4%). Genotype 1 (13.3%) was consisted of the subtype 1a (12.1%) and 1b (1.2%), while genotype 4 (7.4%) comprised the subtype a (6.7%) and b (0.6%). Patients with genotype 5a (0.5%) and 6a (0.4%) were also present. Patients with mix genotypes (3.3%) and undefined genotype (2.4%) were also identified. The frequency distribution of different genotypes is given in Table 1, while Figure 1 illustrates the distribution of genotypes subtypes and mix genotypes in full cohort.

### Association of age, gender, viral load and host serum markers with genotypes

**Figure 2:**
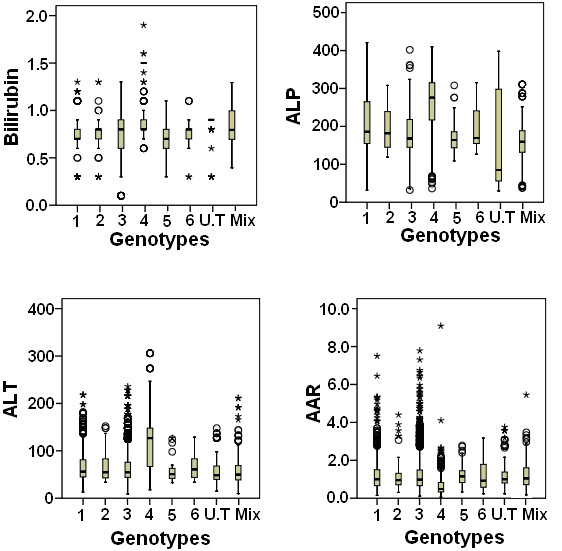
**Box plot of bilirubin, ALP, ALT and AAR for genotypes**. The horizontal line inside each box represents the median, while the top and bottom of boxes represent the 25^th ^and 75^th ^percentiles, respectively. Vertical lines from the ends of the box encompass the extreme data points.

Overall prevalence of genotypes within gender and age groups is given in Table 2. Distribution of genotypes within gender was statistically non-significant (*P *= 0.290), however, incidence of genotypes among age groups (≤ 40 and ≥40) was statistically different (*P *= 0.000). Overall, median value of viral load and host serum markers in each genotype is also given in table 2. Univariate analysis (Table 3) revealed that in three cohorts' serum bilirubin, ALP, ALT and AAR (AST/ALT ratio) were significantly different among genotypes. Figure 2 shows overall median of these four significant variables in different genotypes subtypes and mix genotypes. We observed significant elevated bilirubin, ALP and AST levels, and low AAR value in patients infected with genotype 4 when compared to other genotypes.

**Table 2:** Distribution of genotypes according to gender and age of patients

Variables	Genotypes							
	
	1	2	3	4	5	6	Mix	Undefined
**Gender**								
Male	389	59	2178	226	18	14	94	88
Female	413	54	2109	220	13	12	103	58
**Age groups**								
≤ 40	549	74	2630	304	29	23	124	82
≥ 40	253	39	1657	142	2	3	73	64

**Table 3:** Univariate analysis of biochemical markers in each cohort with respect to genotypes

Dependent Variable	Type III Sum of Squares	Mean Square	*F *value	*P *value
**First cohort**				
Hb Level (mg/dl)	40.762	5.823	1.590	0.133
Bilirubin (mg/dl)	2.736	0.391	11.654	0.000
ALP (IU/mL)	2226420.139	318060.020	93.011	0.000
ALT (IU/mL)	294609.549	42087.078	36.947	0.000
AST (IU/mL)	7017.662	1002.523	.844	0.551
AAR	47.574	6.796	12.561	0.000
Viral load (IU/mL)	3.792 × 10^8^	5.418 × 10^7^	40.886	0.000
**Second Cohort**				
Hb Level (mg/dl)	26.434	4.406	3.399	0.002
Bilirubin (mg/dl)	2.841	0.474	14.248	0.000
ALP (IU/mL)	945658.653	157609.776	35.570	0.000
ALT (IU/mL)	382870.009	63811.668	42.701	0.000
AST (IU/mL)	13693.220	2282.203	2.028	0.059
AAR	28.938	4.823	5.506	0.000
Viral load (IU/mL)	3.020 × 10^6^	5.034 × 10^5^	2.911	0.008
**Third Cohort**				
Hb Level (mg/dl)	4.474	0.639	0.577	0.775
Bilirubin (mg/dl)	4.028	0.575	16.770	0.000
ALP (IU/mL)	1210130.199	172875.743	36.993	0.000
ALT (IU/mL)	772255.261	110322.180	78.063	0.000
AST (IU/mL)	23722.212	3388.887	2.348	0.022
AAR	69.294	9.899	11.921	0.000
Viral load (IU/mL)	3.324 × 10^6^	4.749 × 10^5^	1.927	0.062

### Correlation of serum markers and viral load

**Figure 3:**
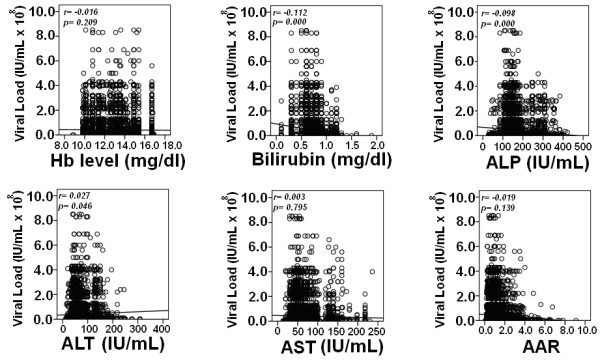
**Spearman correlation of biochemical markers with viral load in overall patients**. Correlation *P *< 0.05 was considered significant. Serum level of bilirubin and ALP were negatively, while ALT was positively associated with viral load in patients infected with HCV.

Correlation of serum markers with viral load in HCV infected patients is illustrated in Figure 3, it shows that viral load has negative correlation with bilirubin (*r *= -0.112, *P *= 0.000) and ALP (*r *= -0.098, *P *= 0.000), while a linear significant correlation of viral load and ALT was found (*r *= 0.027, *P *= 0.046). We observed non significant correlation of serum viral load with Hb level, AST and AAR.

### Association of genotypes and viral load with serum markers among fibrosis stages

The clinical outcomes of the 157 HCV infected patients who underwent biopsy are briefly explained in Table 4. The determination of liver fibrosis showed stage F0-F1 and F2-F3 in 63 patients each, while in F4 or advanced fibrosis leading to cirrhosis there were 21 patients. Genotype 3a and 1a were identified in 135 and 22 patients, respectively.

**Table 4:** Distribution of each variable according to fibrosis stages (n = 157)

Factor	No/minimal fibrosis (F0/F1)	Significant fibrosis (F2/F3)	Cirrhosis (F4)	*P *value
Age	32.7 ± 9.4	40.3 ± 8.4	48.4 ± 7.1	0.000

Sex (M/F)	54/14	46/22	14/7	0.247

Genotype (1a/3a)	12/56	6/62	4/17	0.258

Viral load (IU/mL)	7.8 х 10^6^±1.3 х 10^7^	1.2 х 10^8^±1.9 х 10^8^	2.9 х 10^5^±2.9 х 10^5^	0.000

Hb level (mg/dl)	12.6 ± 1.2	12.8 ± 1.5	12.3 ± 1.2	0.328

Bilirubin (mg/dl)	0.83 ± 0.18	1.06 ± 0.31	1.62 ± 0.31	0.000

ALT (IU/mL)	118 ± 62.4	145.8 ± 76.2	147.5 ± 61.2	0.091

ALP (IU/mL)	81.5 ± 38.1	159.7 ± 79.2	323.8 ± 80.1	0.000

AST (IU/mL)	96.8 ± 63.7	101.5 ± 58.6	155.5 ± 90.6	0.004

AAR	1.03 ± 1.07	0.97 ± 1.06	1.26 ± 0.95	0.522

**Figure 4:**
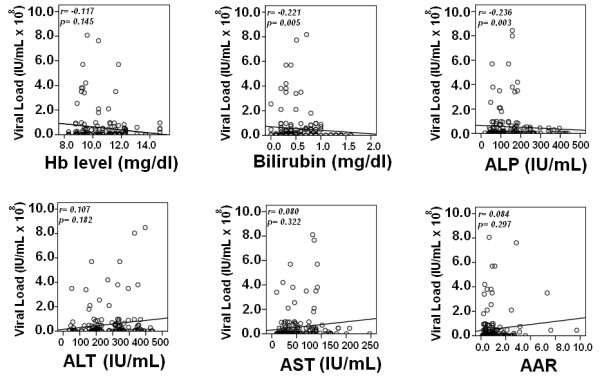
**Spearman correlation of biochemical markers with viral load in patients with histopathological stages**. Correlation *P *< 0.05 was considered as significant. Bilirubin and ALP levels were negatively associated with viral load and were significantly raised in advanced stages of fibrosis.

Fibrosis stages were independent of genotype of the patients. Univariate analysis showed that serum viral load, bilirubin, ALP and AST levels were significantly different among fibrosis stages. Meanwhile, a strong significant negative correlation of viral load with bilirubin (*r *= -0.221, *P *= 0.005) and ALP (*r *= -0.236, *P *= 0.003) was observed as shown in Figure 4.

## Discussion

The basic aim of this study was to find out the association of genotypes and viral load with host age, gender and biochemical outcome and, their association with liver fibrosis progression. Our patient's data showed no significant differences in genotype distribution in relation to gender. Various genotypes, particularly 1, 3 and 4 were equally distributed in gender. However, we observed more HCV dominance in patients with age ≤ 40 years. May be this was due to early assessment of disease. Prevalence of genotypes in our study was: genotype 3 (n = 4287, 70.9%), followed by genotype 1 (n = 802, 13.3%) and genotype 4 (n = 446, 7.4%). Subtypes 3a, 1a and 4a were predominant, whereas mix subtype 4a/5a was also found in some patients (n = 26, 0.4%). Among patients, 2.4% (n = 146) showed untypable genotype. The high prevalence of genotype 3 followed by 1a is according to the previous studies conducted in Pakistan [1,8,19]. Idress et al. 2008 reported increased prevalence of HCV infection due to genotype 4 and 1 without increase in frequency of genotype 3 in various areas of Pakistan mainly Khyber Pakhtoon Khawa (1a, 6.56% and 4, 2.30%) and Balochistan (1a, 25.80% and 4, 4.03%). Our results indicated a 4-5 fold increased prevalence of genotype 4 in Pakistan (1.49% to 7.4%). Moreover, genotype 4 is reported to be associated with liver cirrhosis [20]; there may be risk of increased liver cirrhosis in Pakistan as the percentage of genotype 4 is increasing.

Evaluating the correlation between different clinical markers with genotypes, we observed that four clinical markers AST, ALP, bilirubin level and AAR were significant in all three cohorts' one initial and two validation cohort. This may lead to conclude that these four biochemical markers may be used to differentiate genotypes. However, all genotypes except genotype 4 showed approximately same levels of serum markers bilirubin, ALP, ALT and AAR in patients as shown in Figure 2. We observed significantly high bilirubin, ALP and AST levels in patients with genotype 4 while, the AAR ratio for genotype 4 was considerably lower than other genotypes.

Concerning correlation of viral load with biochemical markers, we found a positive but very weak correlation of ALT with viral load, while a strong negative correlation with bilirubin and serum ALP levels. Weak correlation of serum viremia levels with ALT in our study was in agreement with outcome of *Azzari et al*. 2000 and Murakami *et al*. 2004 that the viral load was independent of ALT activity in HCV [13,21]. Zechini *et al*. 2004 found a relation between HCV viral load and AST. However, we could not observe any correlation between viral load and AST that may be due to poor immune response resulting in uneven AST level and viral load and lead to liver damage [22].

High bilirubin level is usually associated with liver metastases and liver tumor involvement leading to hepatocellular carcinoma and liver cirrhosis by active or non-active HCV or HBV [23]. Bilirubin has been reported as marker of liver injury and to determine the proper dose of interferon in patients with different genotypes [14]. As different genotypes lead to diverse severity of liver disease so the treatment plan of chronic HCV infection with interferon varies with the genotype being treated [24].

Elevated aminotransferases levels act as indicators of liver cell injury [21,25] and are usually predominant in liver cirrhosis with increased ALT levels [26]. We observed mild increase in AST and elevated ALT level in genotype 4 as compared to other genotypes. These results could lead to the confirmation of association of genotype 4 with increased risk of cirrhosis [20].

In our study, patients with genotype 4 reflected high ALP levels as compared to others. Recent studies revealed that the higher levels of ALP are usually associated with liver metastasis, extra-hepatic bile obstruction, primary biliary cirrhosis, intrahepatic cholestasis, infiltrative liver disease, hepatitis, cirrhosis, primary sclerosing cholangitis, hepatic lymphoma, liver abscess, sarcoidosis and congestive cardiac failure. A change in ALP levels greater than 120 U/L can be indicative of advanced disease progression [27-29].

Although high AAR value indicates more chances of cirrhosis, we found low AAR levels for genotype 4 as compared to others leading less chances of cirrhosis; but according to Reedy *et al*. (1998), this test cannot predict significant cirrhosis in patients with chronic hepatitis C [30]. We also observed slightly low Hb levels in HCV patients irrespective of genotype. As HCV is associated with many extra hepatic complications, decline of Hb level with the increase of viral load in HCV may lead to autoimmune haemolytic anemia (AIHA) that can contribute to enhance the liver cirrhosis especially in patients with genotypes 1-4 as the patients with HCV related AIHA have higher prevalence of cirrhosis [31]. Higher ALP and bilirubin levels and mild increase in AST levels in patients with genotype 4 may also lead to cholestatic hepatitis that is a severe form of HCV recurrence after treatment and organ transplantation like liver, kidney and heart [24,32].

We got consistent correlation of ALP, AST, bilirubin, with viral load and genotypes in all three cohorts that is initial cohort and two validation cohorts (Figure 3). We further evaluated the relation of these serum markers with fibrosis stages. As host factors reflect the disease progression, we found that serum bilirubin, ALP and AST gradually increased as fibrosis progressed (Table 4). Fibrosis progression was found to be independent of genotypes, while we found a significant correlation between serum HCV RNA levels and fibrosis stages as shown in Figure 4 (Table 4). As genotype 4 showed different results compare to other genotypes with serum markers we were unable to confirm these results in biopsy samples as we only get biopsy samples of genotype 3 and 1. Our previous pilot study also showed same trend [33]. Kato *et al*. monitored significantly higher HCV RNA level in patients with chronic active hepatitis and cirrhosis compared to chronic persistent hepatitis [31]. Serum HCV RNA level has linear relationship with amount of virus in the liver that is in accordance to De Moliner *et al*. 1998 results [34]. In our study, we observed relatively high viral load in initial fibrosis stages (F0-F1) compared to advance fibrosis stages (F2-F3). An interesting finding was significantly lower viral RNA levels and high bilirubin, AST and ALP in cirrhosis (F4). The increased AST level had been attributed to mitochondrial injury associated with HCV infection and progression of liver fibrosis [35]. In cirrhotic stage (F4) decline in serum HCV RNA levels could be due to reduce number of hepatocytes and advance fibrosis which results in shrinking of liver mass [33]. We also observed a steady increase in serum ALP and bilirubin levels in advanced stage F3 as compared to initial fibrosis (F0-F2) stages that usually observed in significant liver disease or in hepatocellular carcinoma [23,27,28,35,36].

In conclusion, host serum biochemical factors were not found to be dependent on genotype except for genotype 4, where we observed high level of bilirubin, ALP, ALT and lower AAR. In general, viral load showed significant correlation with bilirubin, ALP and ALT. To confirm these results and finding; viral-host association with disease progression was evaluated in liver biopsy samples. We observed that lower viral load and elevated bilirubin; ALP and AST levels are associated with more advanced fibrosis leading to cirrhosis. It is conceivable that serum viral load, AST, ALP and bilirubin levels are suitable factors that can determine liver damage. Although, genotype 4 showed significant variable response to the serum markers, we recommend genotyping assay to find possible association with disease severity and guide about treatment duration and outcomes. Future studies are required to see the relation of the serum markers with genotype 4a infected biopsy samples to find any relation with disease progression.

## List of abbreviations

HCV: Hepatitis C virus; ELISA: Enzyme linked immuno sorbent assay; ALT: Alanine aminotransferases; AST: aspartate aminotransferase; ALP: alkaline phosphatase.

## Competing interests

All authors have no any kind of institutional or financial competing interests.

## Authors' contributions

IB and AW contributed equally to this study. AW, IB and HS designed the study, analyze the data and wrote paper. JFT, SA, GS and SMT performed all lab work. JFT, JS, KS, KH and AS collected and arranged data. All work was performed under supervision of HS. All authors read and approved the final manuscript.

## Authors' information

Bushra Ijaz (M Phil Molecular Biology), Waqar Ahmad (M Phil Chemistry) and Gull S (MSc Biochemistry) are Research Officer; Shah Jahan and Saba Khaliq (PhD in Molecular biology), Javed FT (M Phil, MBBS) is corresponding pathologist, Sawar MT, Asad S and kausar H are Phd scholars, while Dr Aleena Sumrin (PhD Molecular Biology) is Senior Reseach Officer and Sajida Hassan (PhD Molecular Biology) is Principal Investigator at CEMB, University of the Punjab, Lahore
